# Perception of Complementary Medicine and Treatment Adherence as Predictors of Self-Efficacy in Individuals with Chronic Conditions in Mexico

**DOI:** 10.3390/nursrep14020114

**Published:** 2024-06-19

**Authors:** Karina Isabel Casco-Gallardo, Nissa Yaing Torres-Soto, Claudia Nelly Orozco-González, Nancy Griselda Pérez-Briones, José Antonio Guerrero-Solano, Gabriela Maldonado-Muñiz, Claudia Atala Trejo-García, Benjamín López-Nolasco

**Affiliations:** 1 Graduate School of Tlahuelilpan, Academic Area of Nursing, Autonomous University of Hidalgo State, Tlahuelilpan 42780, Mexico; jose_guerrero@uaeh.edu.mx (J.A.G.-S.); gmaldonado@uaeh.edu.mx (G.M.-M.); ctrejo@uaeh.edu.mx (C.A.T.-G.); benjamin_lopez8496@uaeh.edu.mx (B.L.-N.); 2 Department of Medical Sciences, Division of Health Sciences, Autonomous University of Quintana Roo State, Chetumal 77039, Mexico; nissa.torres@uqroo.edu.mx; 3 School of Nursing, Autonomous University of the State of Mexico, Toluca 50180, Mexico; cnorozcog001@profesor.uaemex.mx; 4 “Dr. Santiago Valdés Galindo” School of Nursing, Autonomous University of Coahuila, Saltillo 25160, Mexico; naperezb@uadec.edu.mx

**Keywords:** complementary therapies, treatment adherence, self-efficacy, chronic diseases, diabetes mellitus, type 2, hypertension, obesity

## Abstract

Chronic diseases such as type 2 diabetes mellitus (T2DM), arterial hypertension (HTN), and obesity are significant global health challenges, contributing to millions of premature deaths. In Mexico, these pose major challenges due to limited access to healthcare and inadequate primary care quality. Complementary medicine presents itself as an adjuvant in this context, offering minimally invasive techniques to enhance physical, mental, and spiritual well-being. However, effective treatment adherence is crucial for positive outcomes, influenced by self-efficacy, resulting in persistently low adherence rates—a pressing public health concern. This observational study aimed to explore how perceptions of complementary medicine and treatment adherence predict self-efficacy among individuals with chronic diseases in Mexico. Data were collected from 113 participants with chronic conditions, including T2DM, HTN, and obesity. Participants completed surveys assessing perception of complementary medicine, treatment adherence, and self-efficacy. Statistical analyses, including correlations and regression, were conducted to examine the relationships between variables. The study revealed significant correlations between the perception of complementary medicine, treatment adherence, and self-efficacy. Treatment adherence was positively associated with self-efficacy, while perception of holistic medicine was negatively correlated with self-efficacy. Perception of complementary medicine and adherence to treatment were found to predict 41.9% (*p* = 0.001) self-efficacy. These findings underscore the potential of complementary therapies in enhancing self-efficacy levels, and highlight the importance of holistic healthcare approaches in managing chronic conditions. Further research is needed to better understand these relationships and their implications for healthcare outcomes in Mexico and beyond.

## 1. Introduction

Chronic conditions such as type 2 diabetes mellitus (T2DM), arterial hypertension (HTN), and obesity affect various dimensions (biological, psychological, and social) and constitute the leading cause of death and disability worldwide [1]. This term refers to a set of diseases that are not predominantly acute infections, but which have long-term effects on health, generating the need for prolonged care and treatment [2], highlighting the need for a comprehensive approach by healthcare personnel. It is estimated that annually, these chronic conditions cause the death of 41 million people, representing 71% of deaths worldwide [2]. These fatalities result from inappropriate lifestyles (unhealthy diets, physical inactivity, and tobacco and alcohol use) and genetic factors. These diseases, which pose a significant challenge to healthcare systems, are associated with disability and the loss of productive years of life [3]. In the Americas region, 5.5 million deaths due to these conditions are recorded each year. Globally, more than 15 million people aged 30 to 69 die prematurely from chronic diseases, and over 85% of these deaths occur in low- and middle-income countries. In Latin America, 2.2 million people die from complications of these diseases before reaching the age of 70. The prevalence of overweight and obesity in Latin America is significant, affecting 31.5% and 40.6% of the population over 20 years old, respectively. T2DM has a prevalence ranging from 6% to 17%, while HTN affects 11.7% and 39.7%. In this context, Mexico faces significant health challenges, with high rates of T2DM, HTN, and obesity. Poor quality primary care and inadequate hospitals, along with limited access to healthcare services and low wages for over 70% of the population, may be barriers to timely detection and proper treatment of these chronic diseases [4]. The prevalence of these chronic diseases, especially cardiometabolic conditions like cardiovascular disease, stroke, and diabetes, along with their key risk factors, has shown to be uneven trends across different regions. For example, while cardiovascular disease mortality has decreased in high-income countries over the past few decades, it has increased in low- and middle-income countries (LMICs) such as Mexico. This increase is driven by various factors influenced by environmental, social, political, and commercial determinants of health, such as the shortage of medical personnel, inadequate infrastructure, and disparities in the quality of services between urban and rural areas, among others [5,6].

Despite these challenges, adequate training of healthcare personnel can enable the application of complementary and alternative medicine (CAM) as adjuncts at all levels of care, providing comprehensive care [7]. Additionally, the National Center for Complementary and Integrative Health (NCCIH) defines complementary and alternative medicine as a collection of systems, practices, and products that fall outside the scope of conventional Western medicine, including acupuncture, reiki, homeopathy, herbalism, among others. The distinction between complementary and alternative medicine and conventional medicine is not fixed, implying that these practices may gain wider acceptance over time [8]. These therapies, which are minimally invasive techniques, aim to improve the physical, mental, and spiritual well-being of individuals, promoting self-efficacy to effectively address health issues [9]. An example of the successful application of CAM is provided by San Miguel Borges J. and Martín Aviague [10]. They studied the use of garlic tincture for treating arterial hypertension and headaches. After training nursing personnel, they conducted a descriptive and prospective study with two patient groups: one receiving garlic tincture, and the other receiving conventional treatment. They concluded that a higher percentage of patients experienced symptom relief or resolution more quickly with garlic tincture compared to conventional treatment, contributing to improved health outcomes and reduced economic costs. This can enhance therapeutic adherence and communication with the environment [11].

This comprehensive care, known as holistic care, focuses on the whole person, considering biopsychosocial dimensions to achieve internal harmony. Complementary medicine, as a minimally invasive technique, is presented as an option to provide personalized and holistic care. Although these therapies are designed to complement pharmacological treatment, treatment adherence is crucial for positive outcomes.

The World Health Organization (WHO) defines treatment adherence as the fulfillment of instructions, including the proper intake of medications according to the prescribed dosages and persistence over time. In developed countries, only 50% of chronic patients comply with treatment, turning adherence into a public health issue [12].

In developing countries, it has been noted that Africans and Asians are more prone to not adhering to medication regimens. Nonadherence is particularly high among patients with chronic diseases, with hypertensive patients accounting for nearly 50% of cases. By 2025, the prevalence of hypertension is expected to increase by 30% [13].

The literature suggests that if treatment adherence is effective, patient self-efficacy will also increase [14,15,16]. Self-efficacy refers to the developed and stable capacity of an individual to effectively manage their behavior in response to their medical condition. This component acts as a moderator, stimulant, and protective factor, being fundamental to treatment success [17].

For public health, the use of complementary medicine can provide physical, psychological, and spiritual well-being in combination with conventional treatment and control of chronic health problems [18]. Additionally, they could help individuals achieve the best desirable health status, better adaptation to the environment, proper treatment adherence, and, consequently, empower patients in their illness by improving their self-efficacy, as well as enjoying personal relationships [19]. Firdaus [20] emphasized the positive experiences of chronic disease patients using CAM, driven by economic factors, doubts about conventional medicine, and the influence of family and community support. Therefore, the present research aims to determine the explanatory capacity of a model of perception of complementary medicine and treatment adherence on self-efficacy in people with chronic conditions (T2DM, HTN, and obesity).

## 2. Materials and Methods

### 2.1. Design

This study follows an observational approach with an explanatory correlational design [21] Participants aged 18 or older, diagnosed with chronic conditions such as Type 2 Diabetes Mellitus (T2DM), Hypertension (HTN), and/or obesity for at least six months, and possessing an educational level ranging from primary to postgraduate, were considered eligible. Additionally, individuals who had used some form of complementary therapy for their condition and were attending health centers during the data collection period (January to April 2023) were included.

### 2.2. Sample

Participants were selected through convenience sampling. Among the exclusion criteria were the following: that the person was a minor; and that he/she used some therapy as part of his/her culture and/or religion, since the use of the therapies would be for religious reasons and not for health reasons.

The sample size was estimated using G*power software version 3.1 [22] with a one-tailed test, an effect size of 0.10, an error probability of 1 or alpha of 0.05, and a power of 0.95, with two predictors. Data collection was carried out through the Qualtrics form, the link of which was distributed in primary care clinics in Mexico.

### 2.3. Measures

Sociodemographic Data Sheet: age, gender, marital status, level of education, socioeconomic status, highest occupation, and place of residence.

Holistic Complementary and Alternative Medicine Questionnaire (HCAMQ) [23]: Used to assess patients’ perception of complementary and alternative medicine. It is composed of 11 items in Likert scale format with six points coding as follows: strongly agree (1), agree (2), slightly agree (3), slightly disagree (4), disagree (5), and strongly disagree (6). Items 2, 4, 6, and 9 were reverse-scored, while the rest were positively scored. The scale is composed of two subscales: one with questions about complementary and alternative medicine (2, 4, 6, 8, 9, and 11), and the other about holistic health (1, 3, 5, 7, and 10). A lower score on the HCAMQ indicates more positive attitudes toward complementary and alternative medicine. Internal consistency was measured using Cronbach’s alpha, which was 0.69 in the Latin American population.

MBG Scale (Martin-Bayarre-Grau) [24]: Used to measure treatment adherence. It is composed of 12 Likert-type items with 5 response options. The total possible score is 48, and it is evaluated by proportionally dividing the total points obtained by the patient. Scores between 38 and 48 are considered to be fully adherent, between 18 and 37 are partial adherents, and between 0 and 17 are classified as non-adherent. It has three subscales: Treatment compliance (1, 2, 3, and 4), Personal involvement (5, 6, 8, 9, and 10), and Transactional relationship (7, 11, and 12). This scale, which has a Cronbach’s alpha of 0.88 in Spanish-speaking patients, allows for a quick assessment of responses and classification into three levels of adherence.

General Self-Efficacy Scale [25]: Measures the constant sense of personal ability to handle stressful situations. It is composed of ten items assessed on a four-level scale. Scores range from 10 to 40, and the original scale shows an acceptable internal consistency index of 0.81, according to Baessler and Schwarzer. This scale has three dimensions: magnitude (2, 4, 5, 7); strength (1, 3, 6, 8); and generality (9 and 10).

### 2.4. Statistics

Data analysis was performed using the Statistical Package for Social Sciences (SPSS) ver. 27 (IBM, San Diego, CA, USA). Scale reliability was assessed using the internal consistency index, employing Cronbach’s alpha coefficient. Subsequently, univariate descriptive statistics were calculated, including minimum, maximum, mean, and standard deviation. To verify data normality, skewness and kurtosis tests were applied. In this case, the data did not meet normality parameters, so the Spearman correlation test was used to examine associations between variables of interest. Finally, a multiple linear regression test was conducted using the stepwise method to determine the impact of the variables.

### 2.5. Ethics

The research received approval from the Ethics Committee of the Universidad Contemporánea de las Américas with registration number: EAD0012021-027. To comply with the provisions of the General Health Law of Mexico [26], recognizing the participant as a human being and emphasizing the importance of respecting their dignity, protecting their rights, and ensuring their well-being. The study was classified as minimal risk, and participants were assured they could stop responding at any time if they experienced any health harm during the instrument’s application. Clear and precise informed consent was implemented. Ultimately, this study followed the ethical provisions of the Helsinki Declaration [27] and the principles of justice, beneficence, respect, and non-maleficence of the Belmont Report [28].

## 3. Results

A total of 113 people with chronic diseases participated in this study predominantly females (75.2%) in the age range of 40–49 years (26.5%). The majority reported being married (48.7%) and having studied at the postgraduate level (40.7%). Most participants belonged to the middle socioeconomic stratum (77.9%) and were employees (52.3%). The sample primarily resided in states such as San Luis Potosí, Coahuila, and Guanajuato.

The rest of the sociodemographic characteristics of the study population are presented below (Table 1).

Regarding the participants’ health, it was observed that 68.1% reported the presence of some metabolic disorder, such as overweight or obesity, 28.3% had arterial hypertension, and 18.6% had been diagnosed with T2DM (*n* = 21). Additionally, 32.7% indicated the presence of at least two or more diseases. In total, 44.2% (*n* = 50) of the sample reported currently using some MAC therapy at the time of the survey, and 78.8% (*n* = 89) mentioned using 1 to 3 complementary therapies, the most used being meditation (36.3%), aromatherapy (29.2%), acupuncture (29.2%), and homeopathy (23%).

### 3.1. Scales

Table 2 provides details of the univariate statistics of the scales, revealing a mean score of 26.59 for perception of complementary medicine, indicating a positive perception towards this type of therapy. A mean score of 32.59 was also found for treatment adherence, suggesting partial adherence by the participants. Regarding self-efficacy, a mean score of 32.67 was obtained, indicating a moderately high level of self-efficacy.

### 3.2. Spearman Correlation and Simple Linear Regression Model

Table 3 shows that there is a significant negative correlation of −0.264 ** (*p* < 0.001) between the perception of holistic complementary and alternative medicine and self-efficacy. This indicates that as the perception of holistic medicine worsens, self-efficacy tends to decrease. There is a significant positive correlation of 0.269 ** (*p* < 0.001) between the perception of holistic complementary and alternative medicine and gender. This suggests that perception of holistic medicine tends to vary by gender, with men having a poorer perception of complementary and alternative medicine. There is a significant positive correlation of 0.212 * (*p* < 0.05) between the perception of holistic medicine and the presence of arterial hypertension. This indicates that the perception of holistic medicine tends to be poor in individuals with arterial hypertension. Additionally, there is a significant positive correlation of 0.343 ** (*p* < 0.001) between treatment adherence and self-efficacy. This suggests that as treatment adherence increases, self-efficacy also tends to increase. There is a significant positive correlation of 0.344 ** (*p* < 0.001) between age and the presence of arterial hypertension. This indicates that the likelihood of having arterial hypertension tends to increase with age. Furthermore, there is a significant positive correlation of 0.306 ** (*p* < 0.001) between the presence of arterial hypertension and the presence of T2DM. This suggests that there is a positive association between both conditions. Lastly, there is a significant positive correlation of 0.253 ** (*p* < 0.001) between gender and the presence of T2DM. This indicates that there are differences in the prevalence of T2DM by gender, in this case, in men.

In order to assess whether self-efficacy can be predicted by the perception of complementary therapies and treatment adherence, a regression analysis was conducted using the stepwise method. The results presented in Table 4 indicate that the significance index, statistical power, and effect size are adequate and predict 41.9% (*p* = 0.001) of self-efficacy.

The results obtained from the regression analysis using the stepwise method show that when incorporating the variables of perception of CAM and treatment adherence, they have explanatory power over self-efficacy, demonstrating a higher level of statistical power and elevated effect size. The first index surpasses 0.80, allowing us to assert that the results are relevant for predicting self-efficacy. Additionally, the collinearity indicators VIF (Variance Inflation Factor) exceeding 10 and tolerance values greater than 0.20 indicate high correlations between the factors in the model; and lastly, the Durbin–Watson indicator is above 2.2, which does not allow for data generalization.

## 4. Discussion

This study presents significant findings that highlight the relationship between the perception of CAM, adherence to treatment, and self-efficacy in individuals with chronic conditions. The results reveal that a considerable proportion of participants present metabolic disorders, such as overweight or obesity, T2DM and HTN, highlighting the relevance of exploring complementary strategies in the management of these conditions.

The perception of complementary and alternative medicine in chronic diseases is a topic of interest in various communities, especially in the treatment of T2DM, HTN, and cancer, and several studies have explored the perception of complementary medicine in the context of chronic diseases.

Regarding the perception of complementary therapies, a positive perception was found in general, and it is especially good in women. These findings are consistent with those reported by Hill et al. [29] in their research aimed at documenting the prevalence of the use of traditional, complementary, and alternative medicine in adult cancer patients at a national teaching hospital in Malawi, where female sex was found to be a predictor of the use of traditional, complementary, and alternative medicine over the use of conventional treatment.

Regarding the use of CAM therapies, it was found that the majority of the sample mentioned using more than one complementary therapy, so it was observed that a large part of the sample is resorting to them, with meditation, aromatherapy, acupuncture, and homeopathy being the most common. This is similar with what was mentioned by Villar [30] in his 2016 study, where the most known and accepted alternative therapy was acupuncture followed by phytotherapy, while the most used was phytotherapy. This trend suggests an interest and active search for therapeutic alternatives by participants, possibly motivated by dissatisfaction with conventional treatments or the search for more holistic approaches to their well-being.

Self-efficacy is related in several ways to the use of complementary therapies in chronic diseases. Studies [31] show that a higher level of self-efficacy is associated with better adherence to a healthy diet, including the Mediterranean diet, and the ability to resist unhealthy foods. In addition, higher self-efficacy is associated with lower levels of perceived stress and higher levels of interpersonal support. This is similar to what was found in the present research, as a negative correlation was found between levels of self-efficacy and perception of complementary therapies, suggesting that those with a more negative perception of complementary therapies tend to have lower levels of confidence in their ability to manage their health. The use of complementary therapies is often associated with higher self-efficacy, which may lead to better compliance with therapeutic recommendations and better health outcomes [32].

Moreover, the present study identifies a positive correlation between treatment adherence and self-efficacy, indicating that those who more faithfully follow their treatments tend to have greater confidence in their ability to do so. Like that reported by a study [15] of chronic patients in Michoacán, Mexico, which analyzed the relationship between self-efficacy, perceived social support, and adherence to treatment The results show that self-efficacy was directly related to lower levels of noncompliance and greater perceived social support. In addition, mediational analyses indicated that self-efficacy had a significant direct and indirect effect (through perceived social support and satisfaction with support) on patients’ adherence, specifically in relation to diet and physical exercise.

Regression analysis confirms the importance of perception of MAC and treatment adherence in predicting self-efficacy, suggesting that a positive perception of complementary therapies and increased adherence to treatment may contribute significantly to increasing individuals’ confidence in their ability to manage their health.

While this study provides valuable insights into the relationship between perception of complementary medicine, treatment adherence, and self-efficacy in individuals with chronic conditions, it is not without limitations. Firstly, the cross-sectional nature of the study design limits the ability to establish causality between variables. Future longitudinal studies could provide a more comprehensive understanding of the dynamic interplay between these factors over time. Additionally, the sample size of 113 participants may not fully represent the diversity of individuals with chronic conditions in Mexico, potentially limiting the generalizability of the findings. Moreover, the reliance on self-reported measures introduces the possibility of response bias and social desirability effects. Future research could benefit from incorporating objective measures of treatment adherence and exploring other factors that may influence self-efficacy, such as social support networks.

Despite these limitations, this study underscores the potential of complementary therapies in enhancing self-efficacy levels and highlights the importance of adopting holistic healthcare approaches in the management of chronic conditions.

The effective integration of Complementary and Alternative Medicine (CAM) into conventional healthcare requires a multifaceted approach that considers individualized assessment, healthcare personnel training, integration into care models, ongoing research and evaluation, and the support of appropriate public policies. By implementing these recommendations, healthcare professionals can offer patients with chronic diseases more comprehensive, personalized, and needs-focused treatment options, thereby improving their quality of life and overall well-being. Incorporating these suggestions and considerations into clinical practice can advance towards a more comprehensive, patient-centered healthcare system that leverages the full potential of CAM to enhance the health and well-being of individuals with chronic diseases in Mexico.

Moving forward, efforts to integrate complementary therapies with conventional treatments and to address the identified limitations could further enhance health outcomes and quality of life for individuals with chronic conditions in Mexico and beyond.

## 5. Conclusions

The findings of this study provide a comprehensive view of the relationship between the perception of holistic complementary and alternative medicine (CAM), treatment adherence, and self-efficacy in individuals with chronic conditions. The high prevalence of metabolic disorders among participants underscores the importance of exploring complementary strategies in the management of these health conditions.

The results reveal a mostly positive perception of complementary therapies, especially notable among women, which is consistent with previous research suggesting differential gender interest in these alternative practices. In addition, a wide use of various CAM therapies is observed, indicating a growing interest and active search for more holistic therapeutic options by the participants.

Self-efficacy emerges as a key factor in this equation, showing a significant association with both perception of complementary therapies and treatment adherence. These findings suggest that a positive perception of complementary therapies and increased adherence to treatment may play a key role in strengthening individuals’ confidence in their ability to manage their health.

It is essential to recognize that CAM is not a panacea, and should always be used under the guidance of a qualified healthcare professional. The integration of CAM should be gradual and progressive, taking into account the cultural context and specific needs of each community. Effective communication and collaboration among patients, healthcare professionals, and CAM providers are crucial to ensuring safe and effective care. By addressing current limitations and working towards a more robust integration of CAM, we can move towards a future where healthcare in Mexico is more comprehensive, personalized, and effective, thereby improving the quality of life for millions of people.

In summary, the results of this study provide a solid foundation for understanding the interrelationship between perception of complementary therapies, treatment adherence, and self-efficacy in individuals with chronic conditions. These findings offer valuable insights to inform future public health interventions aimed at improving health management and quality of life in this population.

## Figures and Tables

**Table 1:** Sociodemographic characteristics of study participants (*n* = 113).

Variable	Frequency	Percentage (%)
Age		
18–25	15	13.3
26–30	5	4.4
31–39	26	23.0
40–49	30	26.5
50–59	21	18.6
60–69	12	10.6
70–79	4	3.5
Sex		
Female	85	75.2
Male	28	24.8
Marital status		
Single	38	33.6
Married	55	48.7
Common-law marriage	10	8.8
Divorced/Separated	8	7.1
Widowed	2	1.8
Level of Education		
Elementary school	4	3.5
High school	12	10.6
Bachelor’s degree	51	45.1
Postgraduate (Master’s and Doctorate)	46	40.7
Socioeconomic Status		
Low (barely makes ends meet)	21	18.6
Middle (live comfortably, but without luxuries)	88	77.9
High (live very comfortable with luxuries)	4	3.5
Occupation		
Student	15	13.5
Employee	58	52.3
Retired	8	7.2
Self-employed	22	19.8
Homemaker	8	7.2
State		
San Luis Potosí	22	19.5
Coahuila	21	18.6
Guanajuato	10	8.8
Nuevo León	9	8.0
Estado de México	7	6.2
Tabasco	7	6.2
Others	37	32.7
Health conditions		
Obesity	77	68.1
Type II Diabetes Mellitus	21	18.6
Arterial hypertension	32	28.3
More than one disease	37	32.7
CAM Therapies used		
Meditation	41	36.3
Aromatherapy	33	29.2
Acupuncture	33	29.2
Homeopathy	26	23

**Table 2:** Univariate statistics of the study scales.

Scale	$\bar{x}$	*SD*	Min.	Max.	CI	α
Perception of complementary and alternative holistic medicine (HCAMQ)	26.59	5.72	12	41	25.51–27.66	0.69
Dimension 1: Complementary Medicine	19.51	4.53	7	30	18.66–20.36	0.62
Dimension 2: Holistic Health	7.07	2.31	5	15	6.63–7.50	0.70
Treatment Adherence (MBG)	32.59	9.01	5	48	30.90–34.29	0.86
Dimension 1: Treatment Compliance	11.77	3.17	2	16	11.17–12.36	0.77
Dimension 2: Personal Involvement	13.77	3.97	0	20	13.03–14.52	0.72
Dimension 3: Transactional Relationship	7.05	3.64	0	12	6.37–7.74	0.83
General Self-Efficacy by Baessler and Schwarzer	32.67	5.85	13	40	31.56–33.77	0.89
Dimension 1: Magnitude	6.29	1.46	2	8	6.01–6.56	0.79
Dimension 2: Strength	13.10	2.38	5	16	12.65–13.55	0.69
Dimension 3: Generality	13.28	2.60	5	16	12.79–13.77	0.86

*SD* = Standard deviation, $\bar{x}$ = mean, Min. = minimal, Max. = maximal, CI = Confidence interval, α = Cronbach alpha.

**Table 3:** Correlations between study variables.

Variables	*r*	*p*
Perception of complementary and alternative holistic medicine and self-efficacy	−0.26 **	<0.001
Perception of complementary and alternative holistic medicine and gender	0.26 **	<0.001
Perception of complementary and alternative holistic medicine and presence of arterial hypertension (AHT)	0.21 *	<0.05
Treatment adherence and self-efficacy	0.34 **	<0.001
Age and presence of arterial hypertension (AHT)	0.34 **	<0.001
Presence of arterial hypertension (AHT) and presence of Type 2 Diabetes Mellitus (T2DM)	0.30 **	<0.001
Gender and presence of Type 2 Diabetes Mellitus (DM2)	0.25 **	<0.001

*r* = Spearman correlation, *p* = Significance level. * ≤ 0.05, ** ≤ 0.001.

**Table 4:** Simple linear regression model: treatment adherence and self-efficacy.

Model	F	R^2^	ΔR^2^	B	Standard Error	β	*p*	1-β	F^2^
Self-efficacy	11.691	0.419	0.175	35.430	2.951		<0.001	0.95	0.72
Perception of CAM				−0.338	0.090	−0.328	<0.001		
Treatment adherence				0.191	0.057	0.289	0.001		

F = ANOVA, ΔR^2^ = Adjusted R^2^, B = Unstandardized beta, β = Standardized beta, *p* = significance level, 1-β = Statistical power, F^2^ = Effect size.

## Data Availability

The datasets presented in this article are not readily available due to ethical considerations. The data were collected as part of a research study involving human participants, and strict confidentiality measures were implemented to protect their privacy and confidentiality.

## Public Involvement Statement

No public involvement. Participation in the research is not evidence of public involvement.

## Guidelines and Standards Statement

This manuscript was drafted following the STROBE (Strengthening the Reporting of Observational Studies in Epidemiology) guidelines for observational studies.
